# Papering Over the Cracks in the NHS

**DOI:** 10.34172/ijhpm.2020.160

**Published:** 2020-08-25

**Authors:** Shujhat Khan, Areeb Mian

**Affiliations:** Department of Medicine, Imperial College London, London, UK.

## Dear Editor,

 In the current political climate, there are few things that bring British together people so unanimously. One is the mutual love for the National Health Service (NHS). It is clear that with the current coronavirus disease 2019 (COVID-19) pandemic, individuals within the NHS are going to face an unprecedented level of stress, affecting their physical as well as mental health. The manner in which staff within the NHS have dealt with the crisis has been exemplary and has rightfully led to an increase in appreciation of healthcare professionals by the general population. In response, multiple corporations have started to offer discounts on supplies ranging from food to electrical devices; hotels are offering free accommodation to healthcare workers; and there are even public petitions to allow free car parking. Whilst the first peak has passed, and some normality is returning to services and society. It is important to be prepared for the next wave by understanding and addressing problems that arose previously.

The sheer stress following countless hours of managing overflowing wards, intensive care unit departments, and making difficult decisions that quite literally involve life and death may have had severe harmful effects on mental health.^1^ The situation was worsened by a lack of staff and the situation created by the uncertainty of Brexit. Clearly urgent solutions were required to ease this burden and ultimately allow for improved patient care. Because of the acute nature of this event, a large number of patients within the community were left vulnerable. This is and was particularly relevant for the elderly and the disabled who will likely have less support during this period. Compounded with the years of lack of investment in social care and other community care pathways, the system was bound to buckle under the pressure. In an attempt to cover up the failure to adequately fund the healthcare system and retain the staff therein, the government implored members of the public to volunteer. It highlighted their extreme lack of unpreparedness for a pandemic, despite years of warnings. Nonetheless, hundreds of thousands of individuals offered to volunteer within hours. Unfortunately, many volunteers found that they were not asked to do anything, as community statutory bodies were slow to mobilise a response and by the time the scheme was implemented many communities had organised help themselves. Nevertheless, it was heart-warming to witness selfless acts by members of the public who were tasked with providing food and medicines to vulnerable people, helping with transportation from hospitals, administrative duties within hospitals, and keeping regular contact with those who are isolated.

Perhaps a side effect of these unique circumstances has been the grand spotlight shone on the NHS. It again demonstrates the importance of having a national healthcare system. An elderly patient once, rather eloquently, explained the reason as to why the NHS means so much to the public. He had seen his eldest son grow up with a chronic neurological disorder; his wife suffers from dementia; and he had the unfortunate misfortune of witnessing the death of his first grandchild. Through all of this, the one constant was the remarkable care by the NHS who stuck by him and his family through happiness and grief. This story is not unique. The NHS is a national beacon of pride and seeing the public’s response only serves to highlight how indispensable it is.^2^

 The stress that is placed on the healthcare system is not new, however. It had only been greatly highlighted due to the current crisis, but the pressures will remain even after the resolution of the pandemic. The crisis created an environment where the public felt inclined to help. As lockdown restrictions ease and the burden of cases decreases, people will need to be motivated in other ways to help and the government will have to ensure that the NHS has the resources it needs for the next wave.

It is also important to note that whilst along with the NHS workers, the general public’s mental health would have been severely affected in this crisis. Research from previous pandemics such as the H1N1 influenza in 2009 and severe acute respiratory syndrome (SARS) in 2003, highlighted the immense fear and panic and resultant psychological impact on the populace.^3^ This is likely to have a huge long-term impact on health services for which we are ill prepared.

The lack of leadership displayed by the Department of Health is alarming and clear direction, ideally from an independent body, is required to outline short and long-term healthcare policies irrespective of government cycles. Indeed, years of pay restraints have demoralised the workforce, leading to numerous staff leaving the NHS, only to be filled by locum staff who often charge higher rates.^4^ In response the government has increased the pay of some doctors. Unfortunately, this did not apply to nurses and junior doctors, who now feel snubbed after putting their health, time, and livelihood on the line during the pandemic. Moreover, the unresolved case of Brexit has left many of my colleagues perplexed about how healthcare is going to be affected and, as things stand, will likely lead to further staff shortages.

 It is worth remembering that the NHS was created primarily to treat acute illness and is poorly adapted to manage chronic conditions brought about by an ageing population. In the long term, public health campaigns designed to mitigate chronic diseases are thus urgently required and would be hugely beneficial to ease healthcare burden. As a welcomed side-effect, it will reduce healthcare cost as well. However, in order to address it in the short term, the NHS needs urgently resources it is lacking. This will allow the appropriate development of health and social care systems to support people when they become ill, thereby allowing the shift from the aged biomedical model to the more holistic biopsychosocial model of medicine. Nonetheless, the NHS needs to be fully equipped with the necessary resources to deal with a potential second wave. Volunteers will be an indispensable boon to the NHS, and the United Kingdom as a whole.

## Ethical issues

 Not applicable.

## Competing interests

 Authors declare that they have no competing interests.

## Authors’ contributions

 Both authors contributed significantly towards the article.
